# Perovskite- and Dye-Sensitized Solar-Cell Device Databases Auto-generated Using ChemDataExtractor

**DOI:** 10.1038/s41597-022-01355-w

**Published:** 2022-06-17

**Authors:** Edward J. Beard, Jacqueline M. Cole

**Affiliations:** 1 Cavendish Laboratory, Department of Physics, University of Cambridge, J. J. Thomson Avenue, Cambridge, CB3 0HE UK; 2 ISIS Neutron and Muon Source, STFC Rutherford Appleton Laboratory, Harwell Science and Innovation Campus, Didcot, Oxfordshire OX11 0QX UK; 3 Argonne National Laboratory, 9700 South Cass Avenue, Lemont, IL 60439 USA; 4 Department of Chemical Engineering and Biotechnology, University of Cambridge, West Cambridge Site, Philippa Fawcett Drive, Cambridge, CB3 0FS UK

**Keywords:** Devices for energy harvesting, Energy, Solar cells, Information theory and computation, Cheminformatics

## Abstract

The number of scientific publications reporting cutting-edge third-generation photovoltaic devices is increasing rapidly, owing to the pressing need to develop renewable-energy technologies that address the climate-change crisis. Consequently, the field could benefit from a central repository where photovoltaic-performance metrics, such as the power-conversion efficiency (*η*) are recorded. We present two automatically generated databases that contain photovoltaic properties and device material data for dye-sensitized solar cells (DSCs) and perovskite solar cells (PSCs), totalling 660,881 data entries representing 57,678 photovoltaic devices. The databases were generated by applying the text-mining toolkit ChemDataExtractor on a corpus of 25,720 articles. A multi-faceted evaluation, incorporating manual and automatic methods, was applied to ensure that the data contained therein were of the highest quality, with precision metrics ranging from 73.1% to 95.8%. The DSC database contains 475,045 entries representing 41,680 devices, and the PSC database contains 185,836 entries representing 15,818 devices. The databases are available in MongoDB and JSON formats, which can be queried in Python, R, Java and MATLAB for data-driven photovoltaic materials discovery.

## Abstract (summary)

**Table:** 

Measurement(s)	photovoltaic device parameters
Technology Type(s)	natural language processing

## Background & Summary

It is well documented that the Earth’s primary means of generating energy involves the burning of fossil fuels, a process which produces pollutant materials like CO_2_, resulting in a global-warming effect that could have drastic ramifications on the ecosystem of the planet^1–3^. Emerging renewable technologies pose a promising alternative means of generating energy in a sustainable way, by harnessing the energetic processes that occur naturally in the world, such as the wind (using wind turbines), tides (using hydro-electric dams) and sunlight (using photovoltaic cells). In recent years, within the field of solar power, third-generation photovoltaic cells like dye-sensitized solar cells (DSCs) and perovskite solar cells (PSCs) have shown great promise, but progress in these areas might be significantly accelerated through a more systematic research technique than the traditional process of iterative material-component substitution.

‘Big-data’ approaches are enabling systematic methods for data-driven materials discovery^4–8^. These approaches use existing data about a given application area to predict new functional materials. The data may be computational or experimental in origin. While computational data can be generated *in silico*^9–12^, experimental data may be sourced from scientific documents via text-mining. ‘Chemistry-aware’ natural-language-processing (NLP) tools, such as ChemDataExtractor^13,14^, provide a means of automatically extracting experimental data to afford materials databases which can be systematically explored^15–20^. A process like this has previously been applied to create a UV/vis absorption spectral database of organic compounds for helping drive progress in DSCs, by identifying promising new dye candidates that might be used to achieve high power-conversion efficiencies (PCEs) in these solar cells without the need for expensive inorganic dyes^20,21^. However, the overall efficiency of a third-generation solar cell is also affected by the other materials that make up the different layers of the cell. In order to systematically investigate which architectures work best, there is a need for a database that contains the full macroscopic device structure of a solar cell, not just of one particular material. Such a database could also reveal information about the variation observed in popular structures that have been synthesized multiple times in different studies, to glean information on the underlying variation for that particular architecture. This could provide an important insight into the uniformity and stability of these devices, information that might prove invaluable when considering implementing the large-scale manufacturing of a particular cell.

An effort has already been made to manually create a DSC database, resulting in DSSCDB^22^, which contains details on the materials used in the devices and associated absorption spectra for around 4,000 unique solar cells. This database was created manually by scouring many research articles and the standardization of the results was performed by an expert in the field. Whilst this database is significant as the first of its kind, its expansion is limited by this approach owing to the huge amount of time required to manually identify relevant articles, locate the appropriate properties within the article and use this information to populate the appropriate database fields. As the number of research articles being published is growing at an increasing rate, it is even more difficult to represent the latest research without someone constantly reviewing and adding to the database. The current size of DSSCDB (4,000 entries) also limits its potential applications using machine learning, which can be used to identify useful patterns in the data but it performs better on large datasets. Consequently, there is a need for a similar, larger database that can be created and maintained in a less time-consuming manner. PSCs are also receiving a lot of attention, given their efficiencies are improving at an unprecedented rate, *cf*. their increase in PCE from 13% in 2013 to 25.2% in 2020^23^. It is important to introduce a standardized database for new research fields as early as possible before the data become too sparsely populated, in order to reduce the repetition of work and ensure that the field is being investigated in a systematic way.

To address these points, this paper presents two databases, one for PSCs and one for DSCs, to drive discovery in both of these areas. The DSC database contains 475,045 entries organized into 41,680 records, and the PSC database contains 185,836 entries organized into 15,818 records.

## Methods

This section details the overall pipeline and methods used to create the two databases reported within this paper. It begins by describing the process used to generate the corpora, and to filter out false-positive results. This is followed by a detailed description of the data-extraction algorithm, and the subsequent data-formatting processes used to export the data records into a MongoDB database.

### Generation of the corpus

A corpora of 43,735 articles was generated from the publishers; Elsevier and the Royal Society of Chemistry (RSC). These publishers were targeted as they typically provide articles in the easily parsable HTML format, and provide core metrics and results in the main body of text. Each article was obtained using bespoke web-scraping tools that were designed to adhere to the appropriate text and data mining (TDM) terms and conditions. The articles were targeted using the search mechanism of the web interface provided by each publisher. A corpus of 31,779 articles for the DSC database was obtained this way using the search query ‘dye sensitized solar cell’. For the PSC database, a corpus of 11,956 articles was obtained using the query ‘perovskite solar cell’. Both searches were configured to solely identify documents where these words appeared in sequence. The RSC and Elsevier publishers were explicitly targeted since they provide a markup format that is supported by ChemDataExtractor 2.0 (CDE2)^13,14^, and they provide clear TDM policies.

**Fig. 1:**
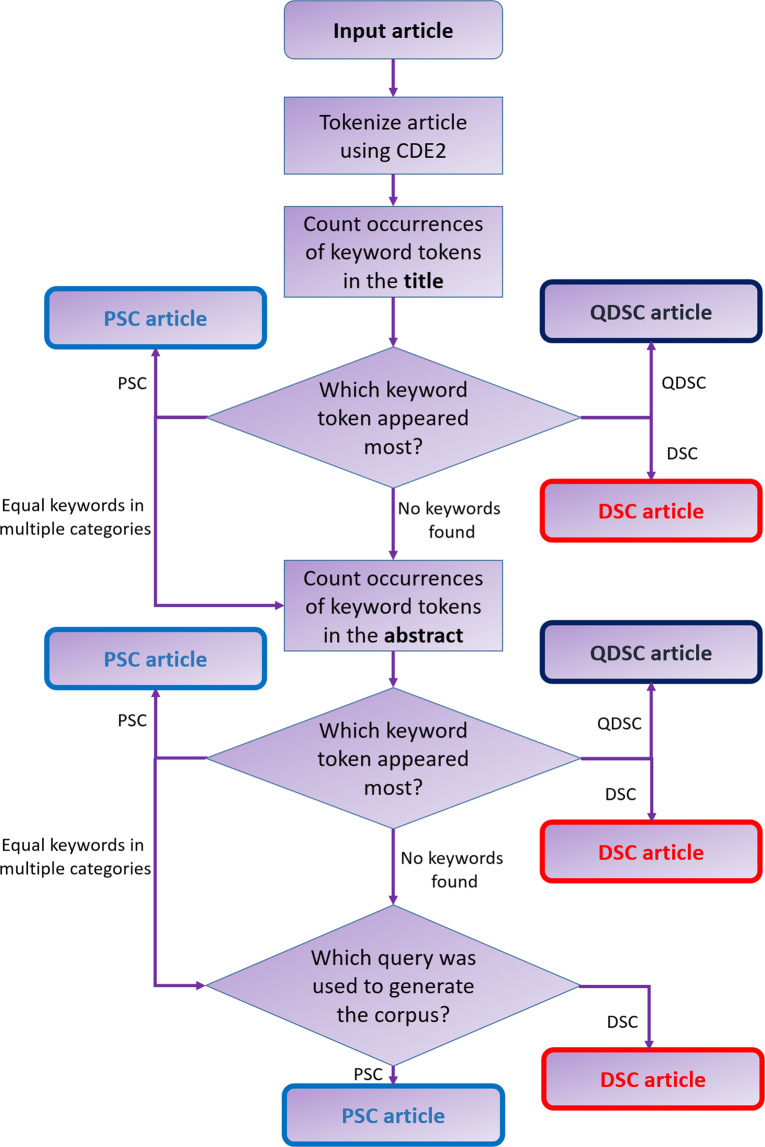
Flow diagram detailing the classification algorithm for filtering the corpus. The various output options are outlined with a bold border, and are coloured in red, blue and indigo according to the classes of dye-sensitized, perovskite and quantum dot solar cells, respectively.

Each corpus was then filtered using the workflow visualized in Fig. 1. This classifies each document by searching through the title and abstract within a document for keywords that indicate the kind of photovoltaic device that is the focus of the article. The focus of the article is not otherwise obvious via text-mining since many papers in the photovoltaics field commonly quote a range of solar-cell device types as introductory material, before proceeding with their new findings on one particular type of solar cell. So a frequency-based classification of ‘dye-sensitized solar cell’ or ‘perovskite solar cell’ is needed to discern whether DSC, PSC or ‘quantum dot solar cell’ (QDSC) is the focus of a given paper. This classification process used the routine in CDE2 that tokenizes the articles to search for these keywords. The category that occurred most frequently in the title was determined to be the class of the document. If no class was found, this routine was repeated on the abstract, and if a classification was still not made at that point, the article was assigned to the default photovoltaic target of the search (DSC for the ‘dye sensitized solar cell’ query; PSC for ‘perovskite solar cell’ one).

Finally, articles without tables were removed before the data-extraction algorithm was executed. This step was taken to avoid wasting computing power in extracting data from articles that will not yield any data records, since the data-extraction algorithm was set to mandate at least one table of photovoltaic properties; otherwise it will not extract anything at all. This was a design decision, made to ensure that the extracted data were as accurate as possible - this was necessary as any one database record can contain up to 23 related properties, each of which must not only be correct but must also be surrounded by the correct values for the device being described. These cognate relationships are more clearly defined in tables, since they are inherently semi-structured, and the successful extraction of these interlinked properties provides a solid foundation upon which the rest of the algorithm can build.

A corpus totalling 25,720 articles remained after these filtering processes, with 17,769 articles used as the input for the DSC database pipeline and 7,951 for the PSC database pipeline.

### Large-scale data extraction

An algorithm was developed to take each article, extract its tabular photovoltaic data and convert it into a series of JSON documents. The algorithm was incorporated into a workflow that allowed it to run in parallel using MPI rank processing, which was implemented using the HPC computer cluster Cooley at the Argonne Leadership Computing Facility (ALCF), IL, USA.

The algorithm was bifurcated into two database goals, one targeted at extracting the DSC data and the other, the PSC data. Each workflow was run separately to create the two databases, which together total 7,059 JSON documents and 57,678 chemical records.

#### General overview

**Fig. 2:**
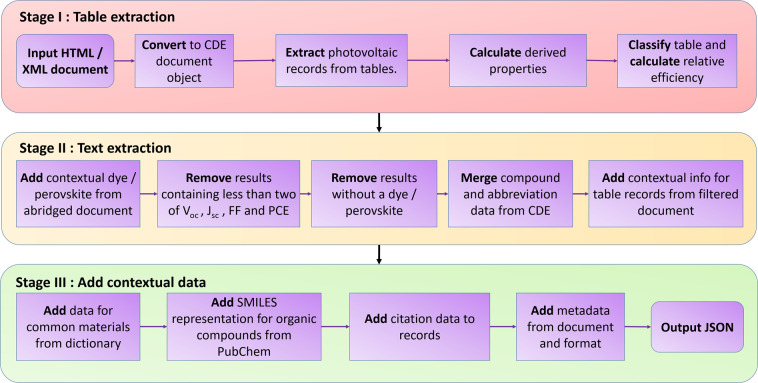
Extraction pipeline used to create database records from a research article.

The photovoltaic records were extracted from the literature using the dsc_db Python library, a new library created specifically for this study, which was designed to extract macroscopic photovoltaic data. This library contains two workflows, one honed to extract DSC records and the other, PSC records. In general, these follow the same operational workflow, although on the occasion where they deviate, the motivation and differences are discussed. The extraction algorithm is shown in Fig. 2.

#### Summary of bespoke changes to ChemDataExtractor 2.0 to tailor it for this work

The dsc_db python library makes use of the ChemDataExtractor-PV (CDE-PV) toolkit, a fork of CDE2 extended to include models for photovoltaic quantities and materials. It also includes the addition of a new, more lenient automatic parser, which only requires a ‘specifier’ rule to identify the property and a ‘raw_value’ rule to extract the value of this property. This addition was made since the automatic quantity parsing provided by CDE2 requires a ‘compound’ field to be detected, which was not a required field for either database.

A range of new models that describe numerical photovoltaic properties were designed to inherit from the pre-defined ‘QuantityModel’ class of CDE2, which automatically handles the conversion of extracted strings to floating point numbers. Each QuantityModel was assigned the pre-defined appropriate ‘Unit’ subclass, and was made to extend existing unit subclasses when appropriate (for example, the ‘area’ unit was defined as the length unit squared). This code snippet illustrates a few models that were used in CDE-PV.

The model parsers for extracting material information (e.g. the dye, HTL, counter electrode) work differently, as they do not extract numerical information; but rather, specific strings that contain material names. They were designed to operate more leniently on tables, such that the presence of a ‘specifier’ phrase in the column header would trigger the extraction of the entire content of the appropriate table cell. When run on sentences, the material name was only populated when the value token was found within a list of common names. This list was manually compiled from a careful examination of some review papers^24,25^, consideration of the options offered by dye and perovskite cell material distributors (Dyenamo, Solaronix, Sigma Aldrich and Greatcell Solar) and expert knowledge of the photovoltaics domain.

An exception to this rule was the sentence parser for the perovskite material, used in generating the PSC database. Halide perovskites are typically reported in Hill notation and are constructed from a particular subset of chemicals in the format ABX_3_, where A typically denotes an organic cation (e.g. CH_3_NH_3_), B is a metal cation from a small subgroup, and X is a halogen. The parser first identifies strings that contain one of the few possible cations for B, and if this is found, it then checks that the string ends with a halogen element, a number, or the variables *x* and *y*. Finally, a check is made to ensure that the string contains three or more uppercase letters; this ensures that at least three elements of the periodic table are used in the perovskite, filtering out any smaller perovskite precursor materials used in the chemical synthesis that might have been incorrectly identified.

#### Operational Workflow for the database auto-generation process

##### Stage I: Table Extraction

This section describes the top layer of the operational workflow of the algorithm described in Fig. 2. The input article is transformed into a CDE-PV document object, automatically converting each section of the paper into a class that has been designed to hold the appropriate data in an easily accessible way (headings, paragraphs, figures, tables etc.).

The table objects are then parsed in accordance with the appropriate photovoltaic model in CDE-PV; for the DSC database, the PhotovoltaicCell model is used, and for the PSC database, the PerovskiteSolarCell model is used. This produces a number of chemical records, where each record describes a unique type of solar cell, and is populated by a number of sub-records that describe different properties of this type of solar cell. These sub-records can contain information about the cell metrology (e.g. active area), device characteristics (e.g. open-circuit voltage) and the material components (e.g. counter electrode). A full list of the supported datatypes is provided in Table 1.

**Table 1:** List of supported properties for each database.

Dye Sensitized Solar Cell Database	Perovskite Solar Cell Database
Open-circuit voltage (${V}_{oc}$)	Open-circuit voltage (${V}_{oc}$)
Short-circuit current density (${J}_{sc}$)	Short-circuit current density (${J}_{sc}$)
Fill factor (FF)	Fill factor (FF)
Power-conversion efficiency (PCE, *η*)	Power-conversion efficiency (PCE, *η*)
Short-circuit current (${I}_{sc}$)	Short-circuit current (${I}_{sc}$)
Power in (${P}_{in}$)	Power in (${P}_{in}$)
Maximum power (${P}_{max}$)	Maximum power (${P}_{max}$)
Active area	Active area
Solar Simulator and Irradiance	Solar Simulator and Irradiance
Series resistance (${R}_{s}$)	Series resistance (${R}_{s}$)
Specific series resistance (${R}_{s}^{sp}$)	Specific series resistance (${R}_{s}^{sp}$)
Charge-transfer resistance (${R}_{ct}$)	Charge-transfer resistance (${R}_{ct}$)
Specific charge-transfer resistance (${R}_{ct}^{sp}$)	Specific charge-transfer resistance (${R}_{ct}^{sp}$)
Reference	Reference
Substrate	Substrate
Counter electrode	Counter electrode
Dye	Perovskite
Semiconductor	Electron-transfer material (ETM)
Redox couple	Hole-transport material (HTM)
Electrolyte	
Dye loading	
Semiconductor thickness	
Exposure-time thickness	

When an extracted sub-record is a quantitative property and contains an interpretable unit, the value is automatically standardized and added to the record. Once all extracted quantities in a photovoltaic record have been standardized, the algorithm attempts to derive additional or duplicate values of the following quantities:

- Solar irradiance
- Short-circuit current density (${J}_{sc}$)
- Short-circuit current (${I}_{sc}$)
- Specific charge-transfer resistance(${R}_{ct}^{sp}$)
- Charge-transfer resistance (${R}_{ct}$)
- Specific series resistance (${R}_{s}^{sp}$)
- Series resistance ($${R}_{s}$$)
- Power in ($${P}_{in}$$)
- Maximum power ($${P}_{max}$$)

These were calculated according to Eqs. (1) to (6).

1 $$Irradiance=\frac{{V}_{oc}\times {J}_{sc}\times FF}{\eta }$$

2 $${I}_{sc}={J}_{sc}\times active\,area$$

3 $${R}_{ct}=\frac{{R}_{ct}^{sp}}{active\,area}$$

4 $${R}_{s}=\frac{{R}_{s}^{sp}}{active\,area}$$

5 $${P}_{in}=Irradiance\times active\,area$$

6 $${P}_{max}={P}_{in}\times \eta $$

where ${V}_{oc}$ is the open-circuit voltage, FF is the fill factor and *η* is the power-conversion efficiency.

At this point, it is worth drawing attention to the active-area property that features in Eqs. (2) to (5). It represents the area of the cell exposed to the simulated sunlight, and it is often reported within the text sections of an article instead of the table itself. In order for these derived properties to be calculated at this stage, an attempt is made to extract the active area from an abridged version of the document called the ‘filtered elements’. These filtered elements are a representation of the article with the sections that describe the introduction, background and conclusion removed. This is achieved by searching for a number of blacklisted keywords in the ‘heading’ objects in the CDE-PV document. This version of the article is also used in the contextual merging sections of the algorithm (see Operational Workflow, Stage II) to ensure that only the sections that focus on reporting new experimental information are scoured for pertinent data, thereby avoiding false positives that might occur in the comparative sections.

Often, research articles in the photovoltaics field describe an experiment using a new material to replace a standard component, such as the dye or counter electrode. To obtain an accurate idea of the impact of this change, the researcher will typically repeat the experiment under the same conditions using the standard component (for example, the N719 dye used in DSCs) to provide a reference point for the result. For such cases, the relative value of the power conversion efficiency (PCE or *η*) for the new versus reference-material-based devices is a very good indication of the significance of the result; yet, it is one that is not explicitly reported in any existing database. To remedy this, a function is executed as the final step of Stage I (Table Extraction) that calculates the normalized value of *η* for each record, with respect to a reference value that is provided inside the table.

This calculation first requires the identification of the specific part of the solar cell whose variation is reported in the table. This is achieved by checking for specific sub-record types that occur for every record in the table; for DSCs, the candidates are the counter electrode, dye and semiconductor; for PSCs, these are the perovskite material, HTL, ETL, and counter electrode. Once the material type has been determined, the appropriate table column is parsed to find a value that matches one of these standard components. The row to which this standard component belongs is classified as the ‘reference device’, and the *η* value in this row is assigned to be the reference for the relative efficiency (${\eta }_{ref}$). Then every other record has its relative efficiency ${\eta }_{rel}$ calculated with respect to this standard value, according to Eq. (7).

7 $${\eta }_{rel}=\frac{\eta }{{\eta }_{ref}}$$

##### Stage II: Text Extraction

The middle layer of the data-extraction algorithm shown in Fig. 2 is now considered. For cases where the chemical names of the certain important structural materials were not obtained from tabular data, a subroutine was employed to extract this information from the table caption or the document. This routine is very important, as a large proportion of articles describe the material composition of the cell in the surrounding text. For the DSC pipeline, this method is first applied to the dye sensitizer; for the PSC pipeline, it is applied to the HTL, the ETL, and the perovskite material. The rules used are:

1. Attempt extraction with a strict parser that only accepts common material names from the table caption.
2. When no result is found, apply strict parser to the ‘filtered elements’ extracted from the document.
3. When no result is found, apply a more lenient parser to the table caption.

The merging routine applies each of these criteria in the order shown, applying the strictest conditions first to ensure that the results are as accurate as possible. When rule 1 does not yield a material, rule 2 is applied, and so on. When more than one material is detected using a particular rule, the most frequently occurring material is determined to be the correct one.

After this, any record that does not contain at least two of the key photovoltaic properties (${V}_{oc}$, ${J}_{sc}$, FF and *η*) is removed. In addition, any records that do not contain a dye or perovskite material are also removed. These strict conditions are applied to ensure that the database is populated with useful data only – it was decided that the minimum requirements for a useful data entry were the presence of a photosensitizer and a few photovoltaic properties.

Next, the entire document is searched for extracted organic compounds and name-abbreviation definitions, using chemistry-aware parsers that are built into CDE-PV. These routines are designed to automatically merge extracted records across the entire document, so that all of the alternative names and acronyms that are used through the article should be merged into a single compound record. Once obtained, these compound data are compared to the organic material sub-records (the dye sensitizer for DSCs, the HTL for PSCs) and if one of the names matches, the surrounding data are merged into the database.

Another round of contextual merging is then initiated, one that merges data from sentences for the properties described in Table 2. Like the previously described merging routine, this attempts to merge values from the table caption, and if this is unsuccessful it looks through the filtered-elements representation of the document. Where multiple values are detected, that which occurs most frequently is assigned.

**Table 2:** Properties applicable for contextual merging from the document.

Dye Sensitized Solar Cell Database	Perovskite Solar Cell Database
Solar simulator (irradiance)	Solar simulator (irradiance)
Substrate	Substrate
Active area	Active area
Semiconductor	Counter electrode
Semiconductor thickness	
Dye loading	
Redox couple	

##### Stage III: Add Contextual Data

The bottom layer of the data-extraction algorithm described in Fig. 2 is then executed. This begins with manually compiling a dictionary of common materials, which is used to enhance the data records. This contains a list of common abbreviations for materials that are often used in the literature. The photovoltaic records are searched for these text strings and, when found, they are populated with the other abbreviations, equivalent chemical names and other useful data. This allows for all records that contain a particular material to be queried in the database, even if the names that are extracted from the document were different. For organic compounds, such as the chemical dye in the DSC or the HTL in the PSC, a SMILES^26^ representation of the molecule is also included in this dictionary.

For organic compounds whose SMILES representations were not resolved in this way, an attempt was made to obtain the SMILES from the extracted name. This check uses pubchempy (https://github.com/mcs07/PubChemPy), a Python interface to the PubChem website which holds chemical information on 93.9 million compounds. For cases where an abbreviation extracted by CDE-PV was merged, this was used to query the database for SMILES strings. If no string was returned, and the record has a compound entry merged earlier in the pipeline, the compound name was used instead in this search. If a SMILES string had still not been resolved by this point, then the raw value of the record was used to query Pubchem.

For perovskite records, a final check is made for records that contain a citation-reference value that has been extracted from a table. For these cases, it is assumed that the data were obtained from a review article, and the extracted citation refers to an article where the specifics on the cell structure can be found. Consequently, any structural information that was extracted from the body of the document of a review article is likely to be unreliable, and it is subsequently removed.

Finally, the metadata are extracted from each CDE-PV document object to be merged with the chemical records. This includes important properties like the digital object identifier (DOI), a unique string that can be used to find the document by appending it to the URL www.doi.org. This is a particularly important field since it allows easy access for a user to any article corresponding to a result of interest, whether to check the validity of the value contained in the database or to trace the exact nature of the experiment. For example, this field could be used in machine-learning pipelines which filter data to a small subset of records for further investigation, to manually cross-validate against the source and remove any incorrectly parsed results.

### Database formatting and upload

The next stage was to convert the fully populated set of 57,678 chemical records into a JSON format. The specific JSON structure is described in detail in the ‘Data Records’ section. Following this, the data are uploaded to two NoSQL MongoDB databases, one for DSCs and one for PSCs. The DSC database is made up of 41,680 chemical records and 475,045 sub-records, whilst the PSC database consists of 15,818 records and 185,836 sub-records. The data inside each database are organised such that the RSC data are stored in one collection, and the Elsevier data in another collection. MongoDB was chosen for the production database since it provides greater flexibility than a standard SQL database, and supports the addition of new fields and quantities if required in future updates. MongoDB also provides support to interface with such a database in most modern computing languages, and it is simple to query the database for specific results and ranges of values.

## Data Records

A static version of the DSC database^27^ and the PSC database^28^ can be downloaded from Figshare. Each database is split into two collections named ‘rsc’ and ‘elsevier’, indicating the publisher from which the data were extracted. All data records within these collections are designed to contain certain mandatory fields, shown here for the DSC database:and here for the PSC database:

Table 3 describes the kind of data contained in each of these fields. The sub-records that can be contained within the ‘device_characteristics’, ‘device_metrology’, ‘(d/p)sc_material_components’ and ‘(d/p)sc_material_metrology’ fields are described within the following code snippet for the DSC database:and for the PSC database:

**Table 3:** Description of data records.

Key	Description	Data Type
Device Characteristics	General properties of the device structure, with no dependence on physical geometry.	Dict
DSC/PSC Material Components	Material components of the solar cell.	Dict
Device Metrology	Numerical data with a dependence on macroscopic attributes of the solar cell.	Dict
DSC/PSC Material Metrology	Numerical data with a dependence on microscopic attributes of the solar cell.	Dict
Table Data	Contextual information about the table from which the record was extracted.	Dict
Device Reference	Citation data extracted from within the table.	Dict
Article Info	The metadata extracted by CDE-PV.	Dict

Quantitative sub-records like ‘voc’ and ‘active_area’ can contain a number of different data types, with all possibilites described in Table 4. Qualitative sub-records like ‘dye’ and ‘htl’ will contain a bespoke set of supported properties depending on the datatype, but this invariably includes a ‘raw_value’ property that contains the extracted text used to refer to the component, and a ‘specifier’ property that contains the extracted text used to identify the property.

**Table 4:** Description of properties that can present in quantitative data sub-records.

Key	Description	Data Type
specifier	Extracted text used to identify the property.	String
raw_value	Extracted text containing the value information from this property.	String
raw_units	Extracted text indicating the units describing this property.	String
value	Numerical values of the property.	List[Float]
std_value	Numerical values of the property converted to the standard unit.	List[Float]
units	Unit data reported using CDE-PVs unit formatting.	String
std_units	Standardized unit data reported using CDE-PVs unit formatting.	String
error	Numerical extracted error of the property.	Float
std_error	Numerical extracted error of the property converted to the standard unit.	Float
derived_value	Numerical values derived using other extracted properties.	List[Float]
derived_units	Unit data reported using CDE-PVs unit formatting, calculated from other extracted properties.	String
derived_error	Numerical estimation of error derived from other extracted properties.	Float
normalized	Dictionary of normalized PCE data with respect to a reference component (only found in some ‘pce’ sub-records).	Dict

The ‘specifier’ and ‘raw’ property information data are retained without modification to provide maximum transparency to the user. Similarly, the ‘article_info’ and ‘table_data’ properties are included to provide contextual insight for every data record. The data records are provided in MongoDB and JSON formats due to the complex nested nature of the dataset.

## Technical Validation

To measure the reliability of the data presented herein, a technical validation was carried out to evaluate the contents of the database from three different perspectives. The most relevant attributes and validation metrics are described and their significance is discussed.

### Manual validation - precision and recall

To ensure that both datasets contain high-quality data, a manual evaluation was performed to estimate the standard precision and recall metrics. These are described by Eqs. (8) to (10).

8 $$precision=\frac{TP}{TP+FP}$$

9 $$recall=\frac{TP}{TP+FN}$$

10 $${F}_{score}=2\times \frac{precision\times recall}{precision+recall}$$

where true positives (TPs) are the correctly extracted outputs, false positives (FPs) are the incorrectly extracted outputs and false negatives (FNs) are results that should have been extracted but were incorrectly omitted. Two data subsets were created to serve the evaluation - one containing articles that were extracted for the DSC database (150 articles) and one that contains articles for the PSC database (200 articles; noting that this sample is 50 articles larger than that used for the database validation, to ensure that the output size of the PSC results would be comparable to the DSC sample, since PSC-based articles were less data rich). Each sample contained an equal number of articles from the RSC and Elsevier, and the individual articles were selected at random.

The articles were then run through the classification filtering routine described in the Methods section, leaving 94 DSC articles and 188 PSC papers. These articles were then run through the data-extraction algorithm, which afforded 34 JSON-formatted articles from the DSC database and 31 from the PSC database. The 34 articles in the DSC sample contained 193 unique photovoltaic records, each representing a solar-cell device. As described in the Data Records section, each record consists of a series of cognate ‘sub-records’ that describe the various properties and materials that make up the solar cell. For the DSC sample, the 193 photovoltaic records contained a total of 1,585 sub-records. The PSC sample contained 241 records and 2,069 sub-records.

In order to assess the accuracy of these data, each sub-record was individually compared against its source in the research article by an expert, and assigned to be a TP or FP; a detailed definition of the evaluation metrics (TP, FP and FN) in this context is described in the supplementary information. Any datum that was not successfully extracted from the table, when it was a quantity supported by the algorithm, was assigned to be a FN. The results for the DSC and PSC evaluation are described in Tables 5, 6 and 7.

**Table 5:** Precision and recall metrics of the sub-records of each sample set.

Description	TP	FP	FN	Precision (%)	Recall (%)	${F}_{score}$ (%)
DSC sample set	1,518	67	75	95.8	95.3	95.5
PSC sample set	1,891	110	68	94.5	96.5	95.5

**Table 6:** Precision and recall metrics of complete records for DSC database sample set.

Description	TP	FP	Precision (%)
Entire PV record	141	52	73.1
Correct dye	162	31	83.9
Correct dye, ${V}_{oc}$, ${J}_{sc}$, $FF$ and $PCE$	162	31	83.9

**Table 7:** Precision and recall metrics of complete records for PSC database sample set.

Description	TP	FP	Precision (%)
Entire PV record	179	62	74.3
Correct perovskite	207	34	85.9
Correct perovskite, ${V}_{oc}$, ${J}_{sc}$, $FF$ and $PCE$	202	39	83.8

To gain a better insight into the accuracy of the extraction, the precision and recall metrics have been broken down into several categories. Table 5 shows these metrics for the DSC and PSC database where each sub-record is evaluated in isolation. These values are extremely high; this could be a result of the focus on the highly structured tabular data that are prioritized by our algorithm. The first rows of Tables 6 and 7 show the precision of photovoltaic records, where every sub-record must be correct for its parent photovoltaic records to be declared a TP. This is an extremely strict condition, and this is reflected in the significantly lower precisions of 73.1% (DSC) and 74.3% (PSC). However, it is worth noting that, in the majority of cases, most of the extracted sub-records are correct – for a given unsuccessful record, often just one or two fields of many were found to be false, so the majority of the data are still valid and usable.

The second rows of Tables 6 and 7 show the results where the dye/perovskite was successfully extracted. The dye and perovskite have been emphasized since these act as the photoactive layer in the solar cell, a role that has one of the greatest effects on the cell efficiency. It is therefore crucial that these values are accurate. The third rows of these tables show cases where the dye/perovskite was successfully extracted alongside the four key photovoltaic properties, ${V}_{oc}$, ${J}_{sc}$, FF and PCE, as these are the most frequently recorded and useful metrics for the characterization of a cell.

In general, the estimated precision for manual human data extraction is considered to be about 80%^29^. It is clear from these results that this value has been exceeded in most cases, and quite significantly for the sub-record condition. Whilst the values for complete records are lower than this reference point (73.1% and 73.6%), the human error would likely be much worse in this case, assuming that: a) each manual datum extraction is independent, b) there is a probability of 0.8 for a successful extraction, and c) taking a standard record containing five sub-records, we can estimate that all records would be correctly extracted in 32.7% of cases (i.e. ${(0.8)}^{5}$). This is likely an underestimate since each case would likely not be truly independent, but it goes some way to highlight that a precision of 73% for a highly cognate dataset is actually very competitive.

### Automated validation

To quantify the accuracy of data extraction over the entire DSC and PSC databases, an automatic validation was performed over all data that contain a ‘solar_simulator’ sub-record with both an extracted value and a derived value. Each derived solar-simulator-irradiance value was calculated from the standardized values of the extracted properties ${V}_{oc}$, ${J}_{sc}$, FF and PCE (*η*) using Eq. (1). The error of derived values was also estimated using the extracted errors of ${V}_{oc}$, ${J}_{sc}$, FF and PCE (*η*), where possible, using standard error-propagation formulae. Where no error was extracted, the number of significant figures was used as as a basis to estimate the uncertainty. The results for the DSC database and the PSC database are shown in Table 8 and Table 9, respectively.

**Table 8:** DSC database - automated comparison between derived and extracted values of solar irradiance.

Description	Correct	Incorrect	% Correct
Within ± 50 Am^−2^	14,329	3,209	81.7
Within derived error	14,498	3,040	77.5
Within derived error or ± 50 Am^−2^	14,541	2,997	82.9

**Table 9:** PSC database - automated comparison between derived and extracted values of solar irradiance.

Description	Correct	Incorrect	% Correct
Within ± 50 Am^−2^	4,870	690	87.6
Within derived error	4,595	965	82.6
Within derived error or ± 50 Am^−2^	4,887	673	88.8

81.7% and 87.6% of extracted and calculated values of the solar irradiance lie within 50 Am^−2^ of each other, showing that the extraction seems to be performing well in the majority of cases. This also implies that the properties ${V}_{oc}$, ${J}_{sc}$, FF and PCE (*η*) and their units were extracted successfully and are standardized. The threshold ± 50 Am^−2^ was chosen as an evaluation metric since it represents a 5% deviation from the standard solar irradiance of 1,000 Am^−2^.

The estimated accuracy from the derived error is slightly lower (77.5% for DSC, and 82.6% for PSC), most likely because of the underestimation of derived error. Where possible, the derived error was calculated from extracted error values; but when this was not possible, the error was estimated on the basis of the number of significant figures reported in the document, which was found to be required for the majority of derivations. This design decision was made under the assumption that each paper would aim to report their results to the same precision as the instrument resolution, but manual analysis of a few examples shows that authors did not strictly adhere to this rule.

It is interesting to note that the accuracy of data extracted for the PSC database exceeded that of the DSC database on both metrics. This is surprising since the parsers for the photovoltaic properties and the logic for calculating derived properties are the same in both cases. One possible cause of this discrepancy might lie in the quality control of publishers that released these data – for example, the PSC database contains nearly equal contributions from Elsevier and the RSC, but DSC database is populated 65% by Elsevier articles. The RSC has taken steps to mitigate inaccurately reported solar-cell device performance metrics following a spate of over-inflated claims by authors which were found to lie in inaccurately reported results. Thereby, the RSC has produced a “Guideline Statement", on “Reporting efficiencies for solar conversion devices" to which prospective authors must adhere before being allowed to publish in an RSC journal^30^.

When including solar-irradiance results that were found to be within the derived error of ± 50 Am^−2^, the DSC database was found to be correct 82.9% of the time, and the PSC database 88.8% of the time. For incorrect cases, the discrepancy in a set of 25 erroneous records was manually evaluated to determine the source. The causes of incorrect extraction were found to be:

- Incorrect units stated in the article, resulting in an incorrect derived irradiance. For example, ${V}_{oc}$ is stated in millivolts when it should have been volts. (6 cases)
- Irradiance was incorrectly stated in the article. (7 cases)
- The extracted irradiance was incorrect. (3 cases)
- The irradiance was derived and extracted correctly, but the discrepancy between the values was larger than expected. This could reflect shortcomings in the experimental method. (7 case)
- Incorrect parsing of the table values, where the specifiers were included inside a cell. (1 case)
- Inconsistencies in the article. (1 case)

### Statistical validation

**Fig. 3:**
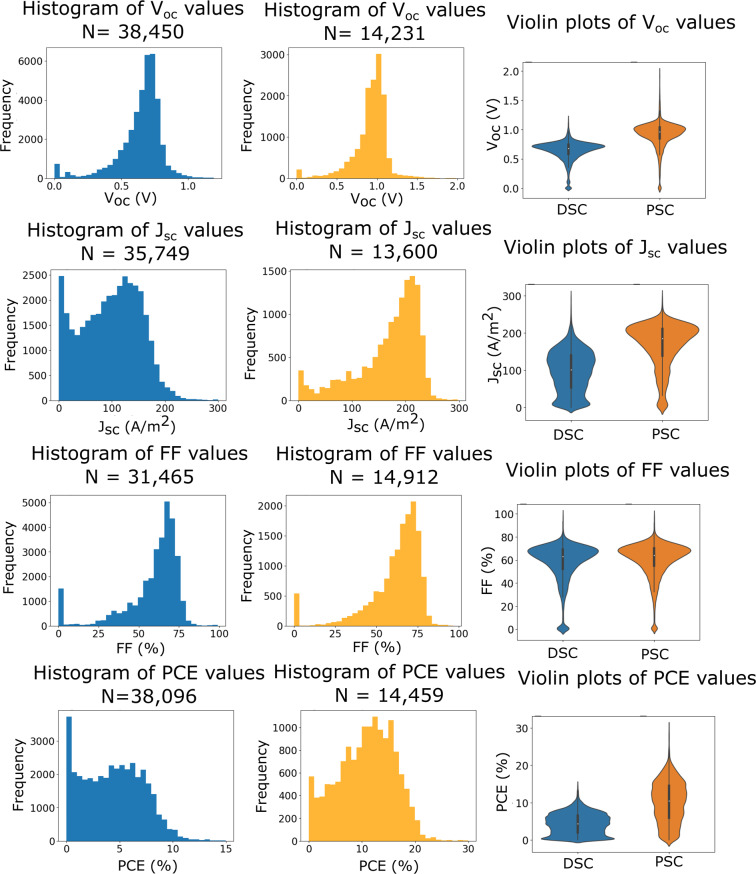
Histograms showing the ${V}_{oc}$, ${J}_{sc}$, FF and PCE distribution for the DSC and PSC databases. First column: DSC database histograms. Second column: PSC database histograms. Third column: Violin plots comparing the distributions for DSC (blue) and PSC (orange).

Histograms and violin plots of the key photovoltaic parameters in each database were generated with outliers removed in order to further validate the data. Outliers were determined by identifying values that lie in physically unfeasible or impossible ranges (e.g. where the fill factor > 100%). Fig. 3 displays some of these data which compare ${V}_{oc}$, ${J}_{sc}$, FF and PCE values, for the DSC and PSC databases.

The DSC histograms appear to exhibit the expected ranges for all quantities. Experimentalists typically aim for specific values of the quantities ${V}_{oc}$ and FF, which is reflected in the modal distributions observed. A slight skew towards the higher values can be observed in both cases, with a sharp drop after the peak; this might be a result of the physical limitations of system architectures. FF acts as a quality control metric to ensure that the cell was optimally designed, and experimentalists aim for solar cells whose FF value exceeds a particular threshold to ensure that their results are indicative of the science and are not caused by some defect in the experimental method that underpins the device physics.

The quantities ${J}_{sc}$ and PCE show a greater spread of values than ${V}_{oc}$ and FF, but they still each contain a broad peak (although this is more pronounced for ${J}_{sc}$). The similarity between these quantities can be explained by Eq. (1). We know that the irradiance is typically constant at 1,000 W/m^2^, and from the uniformly modal distributions of ${V}_{oc}$ and FF, we can assume that these also hold relatively constant between experiments. Therefore, ${J}_{sc}$ and PCE are highly dependent on each other, and a high ${J}_{sc}$ value will be indicative of a high PCE.

For the perovskite database, we observe a similar distribution of ${V}_{oc}$ and FF. The ${J}_{sc}$ and PCE also exhibit a broader spread, and this is more pronounced for ${J}_{sc}$. However, it is clear that there are fewer results in the ${J}_{sc}$ and PCE distributions which exhibit smaller values. This most likely reflects the fact that perovskite solar cells are an emerging field, wherein current research is focussed toward achieving solar cells with the greatest efficiency instead of investigating some of the underlying physical and chemical processes in these devices; in contrast, this is typically undertaken using sub-optimal cell components in the DSC field^31–37^.

For all four PSC histograms, the distributions and peak values are shifted towards higher values. Perovskite solar cells are known for achieving some of the highest performances observed in single-junction third-generation solar cells, surpassing the performance of DSCs, so this is relatively unsurprising. This key difference is emphasized by the violin plots in the third column of Fig. 3, which display the distributions on a common axis.

**Fig. 4:**
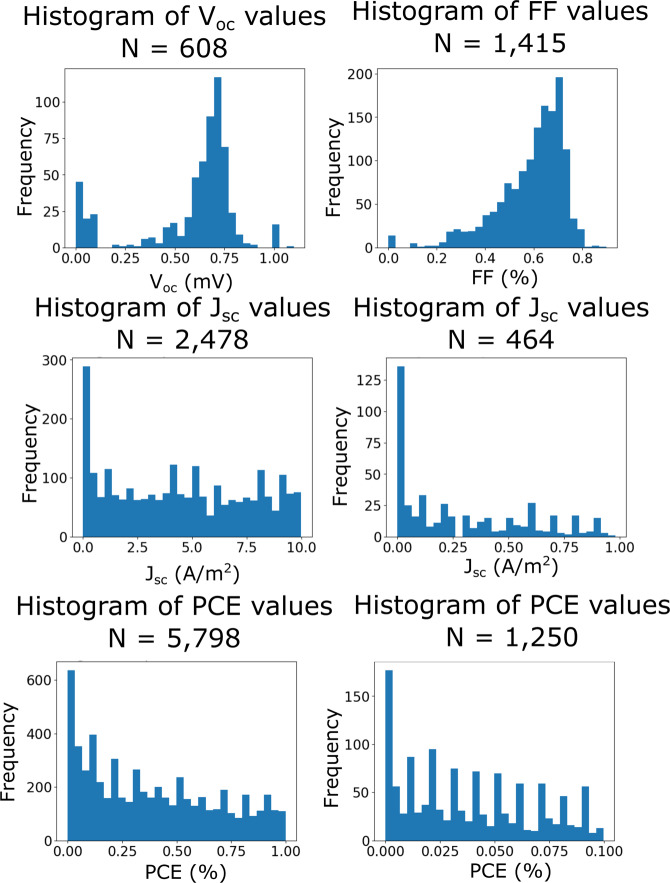
Histograms showing the ${V}_{oc}$, ${J}_{sc}$, FF and PCE distributions for the lower ranges of values in the DSC database. This includes two histograms for ${J}_{sc}$ and PCE with differing resolution.

One feature of note that is observed in the distributions for all properties, for both DSC and PSC data, was the unusually large frequency of the first bin in the histograms; although, the magnitude of this feature varies. Further histograms were created in order to investigate this matter, whereby the range of values was restricted to being equal to or below the size of the first bin, so that the contents were magnified. These histograms can be visualized for the DSC database in Fig. 4.

Analysis of the ${V}_{oc}$ values below 1.2 mV (Fig. 4, top left) reveals a distribution which echos that of the full ${V}_{oc}$ data range, and exhibits comparable statistical descriptors, out by three orders-of-magnitude (for example, the median of the whole dataset was found to be 0.68 V and the median of all values below 1.2 mV was 0.67 mV). This strongly supports the idea that these results are in fact false positives, where the unit provided in the paper was either incorrect or the wrong unit was extracted. Therefore, the decision was made to enhance the dataset by multiplying all results below the 1.2 mV threshold by 1000. A similar correlation was identified from the first bin profile of the fill factor, where the values below 0.1% echo the distribution of the full FF data range. Such cases likely stem from instances where the authors of each article in question added the percentage sign when they actually presented the ratio as a result.

A similar comparison was made for the quantities ${J}_{sc}$ and PCE. However, these appear to show a roughly uniform distribution with distinct sharp peaks for values reported to one significant figure – this is particularly pronounced on the plot for PCE values below 0.1% (Fig. 4, bottom right), where there is a spike for all unitary values that span 0.01 to 0.09. This is not indicative of any underlying scientific pattern, and is therefore considered a feature of the database. These lower values might manifest in experiments where micro-scale physical phenomena, such as charge-carrier recombination, are being investigated – these cases do not typically require the cell to be optimized, and therefore larger values of ${J}_{sc}$ or PCE were not required. Moreover, one likely cause is that these unitary values are rounding errors that were made by the authors of papers that report a result within the resolution limits of of instruments used in solar-cell metrology.

**Fig. 5:**
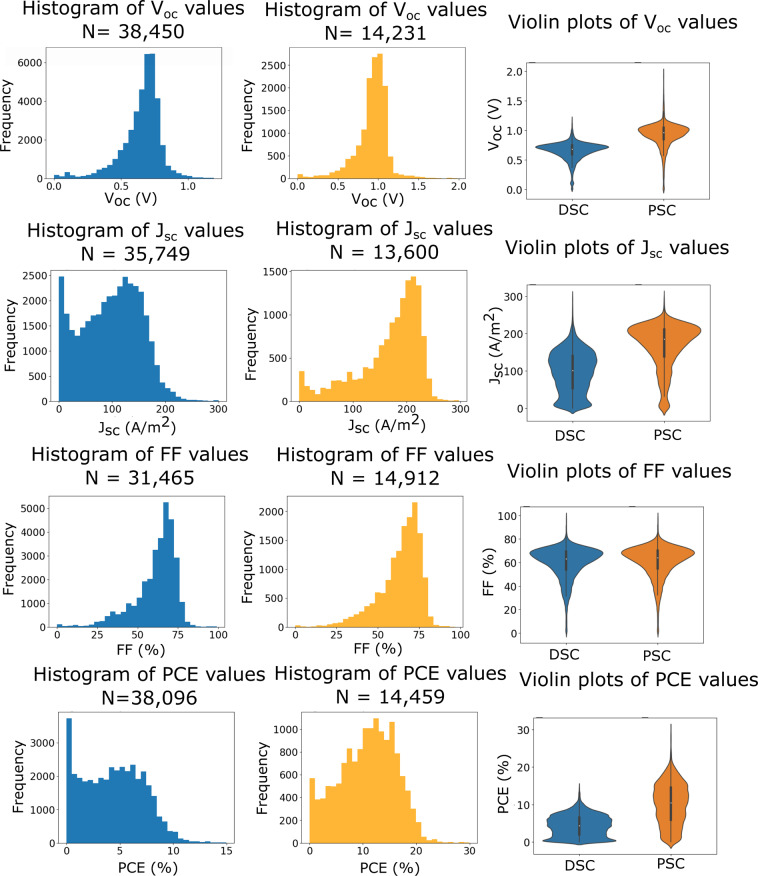
Histograms showing the ${V}_{oc}$, ${J}_{sc}$, FF and PCE distribution for the DSC and PSC databases, after enhancement using data from the first bin where appropriate. First column: DSC database histograms. Second column: PSC database histograms. Third column: Violin plots that compare the distributions for DSC (blue) and PSC (orange).

Analysis of the first bin of the PSC database revealed the same behaviour as the DSC database for all four properties. Accordingly, this database was also enhanced by multiplying the ${V}_{oc}$ and FF results. The updated histograms are shown in Fig. 5.

### Analysis of common materials

**Fig. 6:**
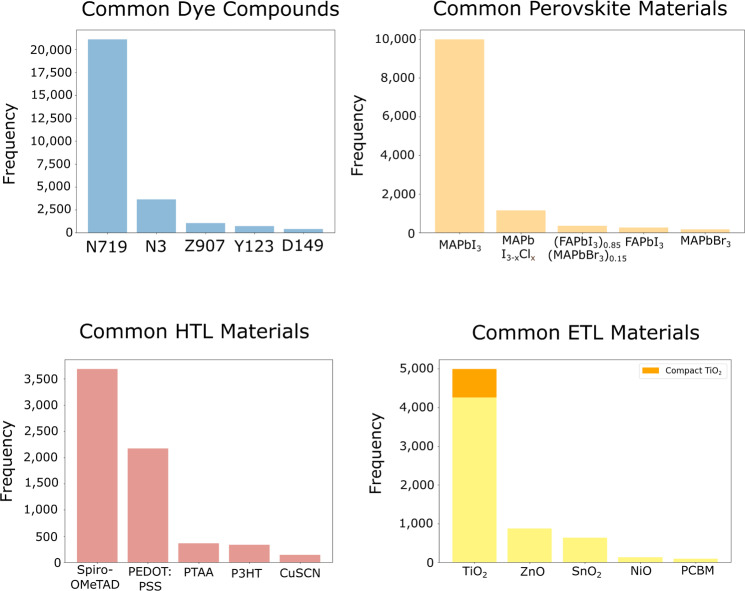
Bar charts for the most common materials in the DSC and PSC databases. Top left: the most common light-absorbing dye compounds. Top right: the most common light-absorbing perovskite materials. Bottom left: The most common hole-transporting materials in the PSC database. Bottom right: The most common electron-transporting materials in the PSC database. The compact form of TiO_2_ mentioned in the bottom right histogram describes a dense, uniform arrangement of TiO_2_ that is typically used to create a thin film in a perovskite solar cell.

A collection of bar graphs was made to investigate the most common materials that are used in the light-absorbing layer of the DSC database (the dye compound) and the PSC database (the perovskite material). These bar graphs are shown in Fig. 6. Similar graphs are alse plotted in Fig. 6 for the PSC charge-carrying materials, the HTL material and the ETL material. Analogous plots about device materials in the DSC database are not shown as they invariably use TiO_2_ as a semiconductor and feature a I^−^/I${}_{3}^{-}$ redox couple as the liquid electrolyte.

It is promising that the most widely used DSC dye (N719) is the most frequently detected, comprising almost half the database at 19,318 occurrences. N719 was one of the first DSC dyes to show particularly high-performance and so it was quick to become an industrial-standard reference dye for DSC research. Consequently, research articles detailing new DSCs report their results alongside a N719 cell that was constructed under the same conditions, for accurate comparison. This may contribute to the huge difference between occurrences of this dye and the next most frequent dye, N3. In turn, N3 is the fully protonated form of N719 and so this is also used commonly as a reference dye. All other dyes present in the histogram are also common abbreviations.

The most frequent values in the perovskite bar chart in Fig. 6 (top right) also reflect those typically observed in experiments. All five of the most common perovskite materials are halide perovskites of the form ABX_3_, or mixtures of perovskites with this structure. As expected, each structure is a hybrid perovskite, with an organic cation of methylammonium (MA) or formamidinium (FA) in site A, lead (Pb) in site B, and a halogen anion in site X. MAPbI_3_ is by far the most commonly reported perovskite.

The reported HTL materials shown in Fig. 6 (bottom left) are also commonly used in PSC structures. The two most frequently occurring materials, Spiro-OMeTAD and PEDOT:PSS, are often used, and they also provide an insight into the structure of the cell; invariably, Spiro-OMeTAD is used in standard n-i-p cell structures, and PEDOT:PSS in inverted p-i-n structures. The ETL materials shown in Fig. 6 (bottom right) are predominantly n-type semiconductors, and the most common of these is TiO_2_. However, it is unusual that the frequency of Phenyl-C_61_-butyric acid methyl ester (PCBM) is relatively low compared to PEDOT:PSS in Fig. 6 (bottom left), as this material is commonly used in inverted cell architectures alongside PEDOT:PSS.

### Comparison with existing database

**Fig. 7:**
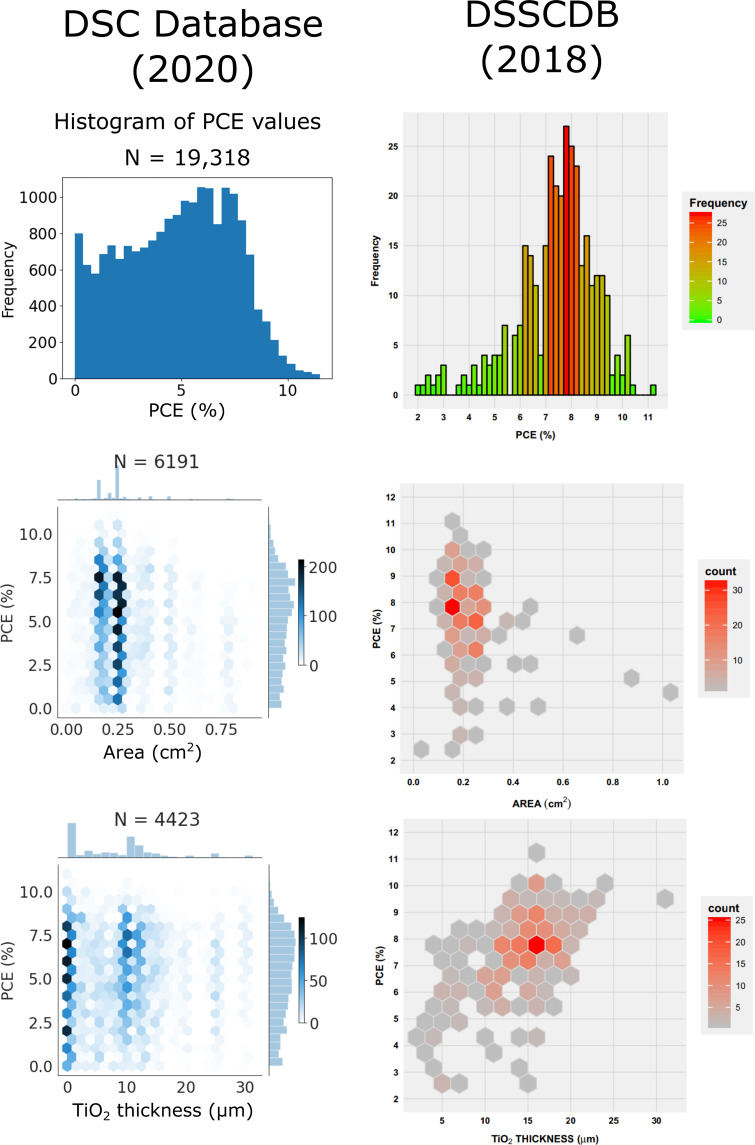
Graphs comparing the new automatically created DSC database (left) to the manually created database ‘DSSCDB’^22^ (right) for all instances of the most common dye, N719. First row: Histograms that show the PCE of all records of the dye N719. Second row: 2-D histograms of the PCE and active area for all records of the dye N719. Third row: 2-D histograms of the PCE and semiconductor thickness for all devices that contain a TiO_2_ layer and the dye N719. The three plots on the right-hand side of this figure are reproduced from Fig. 6 of the paper by Venkatraman *et al*.^22^, with permission under the terms of the Creative Commons Attribution 4.0 International Licence (http://creativecommons.org/licenses/by/4.0/).

A comparison was made between the DSC database reported in this paper and the largest existing publicly available database, DSSCDB^22^. DSSCDB comprises a smaller selection of around 4,000 manually extracted and curated DSC information. The descriptive graphs from Fig. 6 of the original paper about the DSSCDB are presented alongside graphs created from our new DSC database in order to compare the data (see Fig. 7).

The top panels of Fig. 7 show the PCE histograms for our new automatically generated DSC database (2020) (left) and for DSSCDB (right), the manually created database from 2018^22^. Both histograms exhibit a peak at around 7.5% which shows two distinct prongs, although these prongs show greater symmetry for our DSC database. Both figures also show a sharp drop in frequency beyond around 8.5%. However, our new database contains a greater proportion of lower PCE results, which could be indicative of the manner in which the databases were created.

The N719 results produced in the DSSCDB were presumably created from studies that report a device which was designed to perform optimally with N719 as a dye. In our DSC database, a significant number of results will come from experiments from many laboratories across the world where the research is focussed on a new dye, featuring a repeat of the experiment using N719 as the light-harvesting reference dye; the sub-optimal conditions used in many of these laboratories often score lower PCE values. Our DSC database also includes reports that use N719 to investigate the underlying physical processes in DSCs, which typically do not require an optimized cell structure. It is also worth noting that our DSC database contains a much greater number of N719 data entries (19,318 vs. 329), so the DSSCDB may not be fully representative of the literature.

The middle panels of Fig. 7 each show a two-dimensional histogram of PCE against active area for both DSC databases, for cells that contain N719 as a photosensitizer. In both plots, the densest area appears to be between a PCE of 6–8% and an active area of 0.15-0.3 cm^2^. Our DSC database also shows a greater range of values, likely due to the greater amount of data (6,191 instead of 329). It is also apparent that the active areas plotted from our DSC database appear most frequently at two values centered around 0.16 cm^2^ and 0.25 cm^2^. For a DSC with a square active area, side lengths would be 4 cm and 5 cm in these cases, alluding to human tendency to choose round numbers when the exact value of a parameter is relatively arbitrary. The fact that these two most frequent active areas in our DSC database both have PCE values that range from 0.5–11% shows that there is likely little correlation between PCE and active area. This is supported by Eq. (1), since none of the parameters in this equation have any dependence on geometry. This result is also observed in the associated DSSCDB plot shown in Fig. 7.

The bottom panels of Fig. 7 show a two-dimensional histogram that displays PCE against semiconductor thickness, for DSC devices that contain the dye N719 and a TiO_2_ semiconductor. Both graphs display a dense region with a PCE of around 7.5%, although the semiconductor thickness for this region is at 10–12 *μ*m for our DSC database and 12–17 *μ*m for DSSCDB. This histogram for our DSC database also reveals a similar phenomenon to that observed for active area, where there appear to be pockets of data at distinct values of semiconductor thickness (for example, at 10, 12, 20, 25 and 30 *μ*m). These values may reflect a trend in the manufacturing procedures used to create DSC working electrodes, where preferred semiconductor thicknesses prevail for various reasons (for example, scientists tend to target thicknesses with a round number unless there is a good cause to choose a specific target value). Our DSC database also shows an extremely dense region for the lowest bins – these account for DSCs created that implement nanomaterials in their semiconducting layer.

There is no known manually curated PSC database of many experimental values that is publicly available. Therefore a cognate comparison between PSC databases could not be performed.

## Usage Notes

The DSC dataset^27^ and the PSC dataset^28^ are available in JSON and MongoDB formats. To accomodate for the unstructured nature of these data, and to enable the easy addition of new fields to the databases, the information has been recorded in a ‘non-relational’ style as a series of JSON documents and using the MongoDB management framework. There is support for both of these formats in most modern programming languages, including Python, Java, R and MATLAB, which have been highlighted as they represent the most popular platforms used in scientific computing. At the time of publication, the latest documentation containing the syntax for querying a MongoDB database can be found online at https://docs.mongodb.com/manual/core/document/. In addition, there are a number of libraries designed to query a MongoDB database from within these programming languages (such as the ‘pymongo’ library in Python).

## Supplementary information

SI document

## Data Availability

The code used to generate the two databases can be found at https://github.com/edbeard/pv_database. This code makes use of a new library that can be found at https://github.com/edbeard/dsc_db, which defines the logical processes used in the overall data-extraction algorithm. ChemDataExtractor-PV, the bespoke version of ChemDataExtractor, which contains additional models and parsers for the extraction of photovoltaic data, is located at https://github.com/edbeard/chemdataextractor-pv.
