# Supplementary material for: Perovskite- and Dye-Sensitized Solar-Cell Device Databases Auto-generated Using ChemDataExtractor
Source: Sci Data. 2022 Jun 17;9:329. doi: 10.1038/s41597-022-01355-w (PMC9205998; doi:10.1038/s41597-022-01355-w)
Supplement: Supplementary file 1 — SI document [file 41597_2022_1355_MOESM1_ESM.pdf]

Supplementary Information for: Perovskite and Dye-sensitized Solar-Cell Device Databases Auto-generated Using ChemDataExtractor

Edward J. Beard[1, 2] **and Jacqueline M. Cole**[1, 2, 3, 4,*]

[1] Cavendish Laboratory, Department of Physics, University of Cambridge, J. J. Thomson Avenue, Cambridge,4CB3 0HE, UK.
[2]ISIS Neutron and Muon Source, STFC Rutherford Appleton Laboratory, Harwell Science and Innovation6Campus, Didcot, Oxfordshire, OX11 0QX, UK.
[3]Argonne National Laboratory, 9700 South Cass Avenue, Lemont, IL 60439, USA.
[4]Department of Chemical Engineering and Biotechnology, University of Cambridge, West Cambridge Site,9Philippa Fawcett Drive, Cambridge, CB3 0FS, UK.
*corresponding author: (Jacqueline M. Cole: jmc61@cam.ac.uk)

This file contains the definitions of the validation metrics used to calculate precision and recall in the manual evaluation subsection of the technical validation section.

Sub-record evaluation

True positive*:* When the standardized value, unit and error for a particular property or `sub-record' are successfully extracted.

False positive: When any one of the standardized value, unit and error for a particular property or sub-record is incorrectly extracted.

False negative: When any sub-record property is not extracted from a table.

Please note that this definition does not include false positives that were omitted by the contextual merging routine. These data were omitted as they only enhance the key photovoltaic metrics presented in tables and were not considered valid results on their own.

Complete PV record

True positive: When the standardized value, unit and error for all sub-records in a data record are correctly extracted.

False positive: When the standardized value, unit and error for one or more sub-records in a data record is extracted incorrectly.

Note that no false negatives were defined at this stage, as the `first row' property extraction mechanic ensured that some data was extracted for every row of a pertinent table. This logic also applies for the `correct dye' and `correct dye, V_{oc}, J_{sc}, FF and PCE' definitions.

Correct dye / perovskite

True positive: When the common abbreviation or chemical name of a dye / perovskite compound is correctly extracted for a particular record.

False positive: When the common abbreviation or chemical name of a dye / perovskite compound for a particular record is incorrectly extracted.

Correct dye / perovskite, V_{oc}, J_{sc}, FF and PCE

True positive: When, for a particular record, 1) the standardized value, unit and error are correctly extracted for the key photovoltaic properties V_{oc}, J_{sc}, FF and PCE, and 2) the common abbreviation or chemical name of a dye compound is correctly extracted.

False positive: When, for a particular record, any one of the standardized value, unit and error is incorrectly extracted for the key photovoltaic properties V_{oc}, J_{sc}, FF and PCE, or the common abbreviation or chemical name of a dye / perovskite compound is incorrectly extracted.
